# Medical Education

**Published:** 1879-06

**Authors:** 


### Editorial.

### MEDICAL EDUCATION.

For more than twenty years the subject of raising the standard of preparation for the degree of doctor in medicine has been warmly discussed. Most of the plans proposed do not seem to promise assurance of the great object, though they should be faithfully carried out. The length of the course of college instruction; the time of preliminary study before entering college; the season of the year in which the college course is held, and the number of courses requisite to admission to examination, are the plans generally mentioned. These have always impressed the writer with the idea of having emanated from a disposition more to promote the interest of the schools than of raising the standard of preparation in the student. While most of the colleges occupy the winter months for the course, some choose to teach in summer. It is doubtless supposed that the few institutions that adopt the summer session have advantage in point of numbers, and hence the objection urged against them by winter schools. An ostensible reason sometimes given is, that anatomy cannot be successfully studied by practical demonstrations in summer. Even since the art of preserving anatomical material has become so perfect that bodies are kept throughout the entire summer without decomposition, the same opposition exists, proving the objection to be unreal. Another reason is, that the time for completing medical education may be shortened by summer courses about six months; and as the lengthening of student life seems to be the desideratum in all the proposed improvements, they are opposed. Everything proposed by a majority of the schools looks to an extension of the term of study, and most other propositions are now given up for an increase of the number of courses.

The late college convention, which assembled in Atland on the 2d of May, was called the year before by the college association to discuss this subject, and particularly the question of increase in the number of courses. The leading spirits in that convention seemed determined that no other question should be discussed. The committee appointed by the convention, after the organization, to report matter for discussion, refused utterly to report any other question for consideration by the committee except that of increasing the number of courses, and more perfect literary education. The proposition to consider a plan acknowledged more effectual and certain to insure higher grade of preparation was rejected.

Now, if the object be really to protect the public and profession against uneducated practitioners of medicine, by all means the plan which will insure this result, if any such plan is known, should be adopted. The most zealous advocate of extending the term of study, frankly acknowledged in the above mentioned committee that the separation of licensing from teaching is the most effectual, and in fact the only certain mode of accomplishing the great object; and yet there was no disposition in them to advocate it, on the ground, as then expressed, that the time had not arrived for such a proposition to be adopted.

As a teacher, I would expect personally to be pecuniarily benefitted by compelling the student to take an additional course and thereby pay one-third more of fees, rather than compelling him, without regard to time of study, to make a test of preparation before those who have no relations or sympathy with him personally; but if thorough preparation be my prime desire, I cannot object to a special board for his examination, whose only business with him is to test his preparation.

The practicability of having an independent board in each State for the examination of all who desire to practice medicine, has been proven already in Texas and other States. Legal authority, in the form of a college diploma, is now readily obtained from certain chartered institutions, and whether the profession recognizes it or not, the effect upon the public is the same; and in order to secure protection against uneducated pretenders, who never made medicine a study, it is necessary to include all in the call to a rigid examination. What have those to fear who are prepared to practice medicine? Certainly all should be willing to undergo this slight inconvenience, for protection of the public and themselves against men whose only qualification consists in the act of deceiving the patients of educated physicians into the acceptance of irrational and sometimes injurious treatment. Those who are already authorized by law to go forth as physicians may have to be permitted to continue, whether qualified or not, but the foundation can now be laid for a more heathy state of the profession in future. Indeed, no wrong would be done any one if all should be subjected to examination, without regard to previous authority. If practicing physicians generally could make up their minds to take this inconvenience upon themselves, of course charlatans, impostors and interlopers could have no just cause of complaint; and if the profession generally would consent to this, it strikes us as the easiest mode of getting rid of humbugs and impostors in medicine. The profession, in States which have adopted this plan, have subjected themselves, and feel well repaid, no doubt, in the exodus of hundreds who feel their own imperfections.

In the board by which it is proposed to purge the profession, we do not suggest a mere sinecure office which entitles members to a fee from every one that will buy a license, but a board of sworn physicians, not dependent for their salary upon fees thus obtained, and subject to suspension and removal for failure to perform their prescribed duty. The examinations should be made in writing filed for inspection, so that justice to parties examined may be always insured, by having the examination open to criticism and attack.

It is certainly true that no particular term of study will insure preparation, and if examinations are made anything more than a farce, there is no use whatever in requiring any specified time. One student will require four years to accomplish what another may do in two, and great injustice would be practiced upon the studious, without insuring preparation of the indolent, by compelling him to take the additional course.

Laying all other considerations aside, it is the duty and interest of all devotees of the medical profession to accept that plan which not only insures the thorough preparation of medical students, but which will also rid the country of professional interlopers for all time to come. It is a duty we owe the profession and those who are to succeed us in its practical duties.

